# Complete Genome Sequence of *bla*_IMP–6_-Positive *Metakosakonia* sp. MRY16-398 Isolate From the Ascites of a Diverticulitis Patient

**DOI:** 10.3389/fmicb.2018.02853

**Published:** 2018-11-22

**Authors:** Tsuyoshi Sekizuka, Mari Matsui, Tomiyo Takahashi, Michiko Hayashi, Satowa Suzuki, Akihiko Tokaji, Makoto Kuroda

**Affiliations:** ^1^Pathogen Genomics Center, National Institute of Infectious Diseases, Tokyo, Japan; ^2^Antimicrobial Resistance Research Center, National Institute of Infectious Diseases, Higashimurayama, Japan; ^3^The Public Health Institute of Kochi Prefecture, Kōchi City, Japan

**Keywords:** *Kluyvera*, *bla*_IMP-6_, carbapenemase, IncN, *Metakosakonia*

## Abstract

A novel species of carbapenemase-producing Enterobacteriaceae (CPE) was isolated from a patient diagnosed with sigmoid colon diverticulitis. At first, laboratory testing suggested it was *Klebsiella oxytoca* or *Pantoea* sp.; however, a complete genome sequence of the isolate, MRY16-398, revealed that it could be novel species, most similar to [*Kluyvera*] *intestini*, of which taxonomic nomenclature is still under discussion. Orthologous conserved gene analysis among 42 related bacterial strains indicated that MRY16-398 was classified as the newly proposed genus *Metakosakonia*. Further, MRY16-398 was found to harbor the *bla*_IMP-6_ gene-positive class 1 integron (In722) in plasmid pMRY16-398_2 (IncN replicon, 47.4 kb in size). This finding implies that rare and opportunistic bacteria could be potential infectious agents. In conclusion, our results highlight the need for continuous monitoring for CPE even in nonpathogenic bacteria in the nosocomial environment.

## Introduction

Antimicrobial resistance (AMR) is a global issue linked to increased and often unrestricted antibiotic use in the clinical settings, which leads to the dissemination of carbapenem-resistant Enterobacteriaceae (CRE) in health care facilities (World Health Organization, 2017). Carbapenemases comprise three of the four Ambler classes as follows: Class A (*Klebsiella pneumoniae* carbapenemases, KPC, some variants of Guiana extended-spectrum β-lactamases, GES), Class B (metallo-β-lactamases, MBL including New Delhi metallo-β-lactamases, NDM, Verona integron-encoded metallo-β-lactamases, VIM, and imipenemase, IMP), and Class D (OXA-48-like carbapenemases) (Logan and Weinstein, 2017). These carbapenemase-producing Enterobacteriaceae (CPE) have the potential to facilitate the widespread transmission of antimicrobial resistance genes (ARGs) via mobile genetic elements through processes including natural competence, transformation, and plasmid transconjugation that can occur in any environment (Kelly et al., 2017; Rozwandowicz et al., 2018).

The widespread detection of CPE is an emerging issue with potentially serious public health implications; further, the distribution of the most common carbapenemase genes in Enterobacteriaceae occurs in a country- and region-specific manner (Logan and Weinstein, 2017). In Japan, IMP is the most predominant type of carbapenemase among clinical CPE isolates (Koyano et al., 2013; Ohno et al., 2017; Yamamoto et al., 2017). VIM, OXA-48-like, KPC, and NDM carbapenemases are detected at low frequencies in Japan, whereas KPC and NDM are predominant in China and OXA-48-like and KPC are the predominant types of carbapenemases in Europe and United States, respectively.

Most CRE/CPE infections occur in hospitals, with major outbreaks at long-term care facilities and affecting patients with severe medical conditions under long stays for clinical management (Grabowski et al., 2017). There are a number of factors that predispose individuals to infections by CRE and other multi-drug resistant Enterobacteriaceae, including extended-spectrum β-lactamase (ESBL)-producers. Indeed, healthy carriers of CTX-M-type ESBL-harboring bacteria represent major public health concerns, because the carriage rates are on the rise, particularly in South East Asia and Eastern Mediterranean regions. Further, carriers from these regions could potentially spread these bacteria to other communities (Woerther et al., 2013).

Exposure to AMR bacteria can cause serious infections in patients with risk factors such as empirical antimicrobials, advanced age, immune-suppression, admission to the intensive care unit, mechanical ventilation, transplantation, and prolonged hospital stay (Gasink et al., 2009). Early intervention, through the administration of effective antimicrobials to such high-risk group patients, must be achieved to prevent death. In addition, a recent systematic review identified a prevalence of 0–29.5% for community-associated CRE, suggesting that the early detection of CRE-carriers among hospitalized patients upon admission to long-term care facilities might help to prevent nosocomial outbreaks and control the limited distribution of such emerging public health threats (Kelly et al., 2017).

Generally, *Klebsiella*, *Escherichia coli*, *Enterobacter*, *and Citrobacter* have been reported as the main contributors to the nosocomial transmission of CPE (Hrabak et al., 2014; Goodman et al., 2016; Kwong et al., 2018). Other opportunistic pathogens among Enterobacteriaceae species can acquire carbapenemase genes through plasmid transmission from main CPE contributors. *Kluyvera* is a group of gram-negative rod-shaped bacteria and is a member of the Enterobacteriaceae family; the genus contains four species, namely *Kluyvera ascorbata*, *Kluyvera cryocrescens*, *Kluyvera georgiana*, and *Kluyvera intermedia*, which have all been found in humans (Farmer et al., 1981). *K. ascorbata* and *K. cryocrescens* were reported as potential pathogens that are associated with sepsis, bacteremia, catheter-associated urinary tract infections, pyelonephritis, and intraabdominal symptoms in immunocompromised hosts (Karadag Oncel et al., 2015; Yoshino et al., 2016).

Here, we report a novel IMP-6-producing isolate of *Metakosakonia* sp., namely strain MRY16-398, from a clinical specimen (ascites), and determined the genomic features of this carbapenemase-producing species.

## Materials and Methods

### Ethics Approval

The study protocol was approved by the ethics committee of the National Institute of Infectious Diseases in Japan (Approval No. 642, 11/Dec/2015). It was conducted according to the principles of the Declaration of Helsinki, in compliance with the Law Concerning the Prevention of Infections and Medical Care for Patients of Infections of Japan; the ethical committee waived the need for written consent regarding the research of bacteria isolates; the personal data related to the clinical information were anonymized, and we do not request written consent for all patients suffering from bacterial infections.

### Bacterial Strains and Identification

Upon admitting a patient complaining of acute abdominal pain, abdominal computed tomography (CT) scanning showed a diverticulum in the descending and sigmoid colon, resulting in the diagnosis of sigmoid colon diverticulitis. A summary of laboratory data for the patient is shown in Supplementary Table S1. Empiric antimicrobial treatment with cefmetazole (0.5 g twice per day) was administered, and the volume of ascites was decreasing at 5 days from onset.

The *Metakosakonia* sp. MRY16-398 strain was isolated from the ascites of a patient with sigmoid colon diverticulitis in 2015 in Japan. The isolate was identified as *Klebsiella oxytoca* at the hospital microbiology laboratory using BD Phoenix (Becton Dickinson) with low reliability. Further phenotypic tests were performed using API 20E (bioMérieux) and Lysine-Indole-Motility Medium (Nissui, Tokyo Japan). Matrix-assisted laser desorption ionization-time of flight mass spectrometry (MALDI-TOF MS)-based identification was conducted with a MicroFlex LT mass spectrometer (Bruker Daltonik), and analyzed using MALDI Biotyper software (Bruker Daltonik).

### Antimicrobial Susceptibility and CPE Screening Tests

Antimicrobial susceptibility was investigated by broth microdilution using the MicroScan Neg MIC 1J panel (Beckman Coulter) and Etest (bioMérieux) according to manufacturers’ instructions (Clsi, 2018). Boronic acid, clavulanic acid, and sodium mercaptoacetic acid (SMA) were used as inhibitors for double-disk synergy tests (DDSTs) to identify AmpC-types and KPC-types, as well as extended-spectrum and metallo-β-lactamases, respectively. Carbapenemase production was assessed by performing a Carba NP test, as described previously (Nordmann et al., 2012). PCR testing was subsequently performed for potential CPE using a specific primer-pair for the following types of β-lactamase-encoding genes: *bla*_IMP_ (Shibata et al., 2003), *bla*_V IM_ (Shibata et al., 2003), *bla*_OXA-48-like_ (Poirel et al., 2004), *bla*_KPC_ (Bradford et al., 2004), and *bla*_NDM_ (Segawa et al., 2017).

### Plasmid and Chromosome DNA Analysis With Short-Read Sequencing

Plasmid DNA was separated from chromosomal DNA by S1 nuclease-digestion followed by pulsed-field gel electrophoresis. Visible plasmid DNA and chromosomal DNA bands were extracted from the gel and purified using the ZR-96 Zymoclean gel DNA recovery kit (Zymo Research, Irvine, CA, United States). A DNA sequencing library was prepared using the Nextera XT DNA sample preparation kit (Illumina, San Diego, CA, United States) and was sequenced using an Illumina MiSeq and NextSeq 500 for plasmids and chromosomes, respectively. Sequencing reads (plasmid: 2 × 300-mer, 140 × median coverage; chromosome: 2 × 150-mer, 99 × median coverage) were assembled into contigs using the A5-MiSeq pipeline (Coil et al., 2015). Plasmid replicon typing was performed using the curated PlasmidFinder database at the CGE website^1^ (Carattoli et al., 2014).

### Whole-Genome Sequence (WGS) Analysis With Long-Read Sequencing

Genomic DNA from the isolated strain was purified by collecting cells from a 5-mL overnight culture grown in TSB broth. The cell pellet was resuspended in 500 μL of TE10 [10 mM tris (pH 8.0) and 10 mM EDTA] supplemented with 500 μL phenol/chloroform, and the cells were subsequently lysed by bead-beating for 10 min in ZR BashingBead lysis tubes (Zymo Research, Irvine, CA, United States) attached to a vortex adapter (MO BIO Laboratories, QIAGEN, Carlsbad, CA, United States). After centrifugation at 10,000 × *g* for 5 min; the upper phase was further purified using a Qiagen DNA purification kit (Qiagen, Germany).

The complete genome sequence of the strain was determined using a PacBio RSII sequencer for long-read sequencing (SMRT cell v3 using P6C4 chemistry; insert size, approximately 10 kb). Purified genomic DNA (∼2.0 μg) was used to prepare a SMRTbell library using a SMRTbell template prep kit 1.0 (PacBio, Menlo Park, CA, United States) according to manufacturer’s instructions. The obtained raw polymerase reads were analyzed using the HGAP v3.0 pipeline based on Celera *de novo* assembler and Quiver polishing scripts (Chin et al., 2013).

*De novo* assembly was performed using HGAP 4 of SMRT Link Analysis v. 4.0.0.190159 and circulator version 1.5.3 (Chin et al., 2013). Error correction of tentative complete circular sequences was performed using Pilon version 1.18 with Illumina short reads (Walker et al., 2014). Annotation was performed using DFAST (Tanizawa et al., 2018) with databases as follows: DFAST default database, ResFinder database (Zankari et al., 2012), Bacterial Antimicrobial Resistance Reference Gene Database (PRJNA313047), and Virulence Factors Database (Chen et al., 2012). Circular representations of complete plasmid sequences were visualized using GView server (Petkau et al., 2010).

### Comparative Genome Sequence Analysis

All publicly available draft genome sequences were searched based on 16S rRNA gene homology, comparing them to that of the MRY16-398 strain, and 41 entries were retrieved from the NCBI genome database (see Supplementary Table S2). Among those 42 strains, orthologous core-gene sets were extracted using a nucleotide homology search with a threshold ≥80%, resulting in the identification of 479 core-gene sets (see Supplementary Table S2). Using these core-gene sets, core-gene phylogeny was generated using the maximum-likelihood phylogenetic method with FastTree v2.1.10 (Price et al., 2010). To construct a pairwise amino acid homology distance matrix, all amino acid sequences were compared pairwise, against each other, for each genome using the BLASTP program, which was followed by the calculation of average identity scores and standard deviations (Supplementary Table S3).

### Nucleotide Sequence Accession Numbers

The complete, annotated genomic sequence of *Metakosakonia* sp. MRY16-398 was deposited in a public database (accession numbers: chromosome, AP018756; pMRY16-398_1, AP018757; pMRY16-398_2, AP018758). The short- and long-read sequences for DNA-Seq were deposited in the DNA Data Bank of Japan (BioProject PRJDB7098, BioSample SSUB009772, DRA accession DRA007011).

## Results

### Bacterial Identification of Metakosakonia sp. MRY16-398

A potential CPE, designated as strain MRY16-398, was isolated from the ascites after abdominocentesis. The isolate was identified as *Klebsiella oxytoca* at the hospital laboratory using BD Phoenix (Becton Dickinson) with low reliability, whereas API20E testing indicated the isolate should be a *Pantoea* sp. instead of *K. oxytoca.* This isolate was negative for lysine decarboxylate activity and showed weak motility, which indicated that the isolate was not *Klebsiella.* MALDI-TOF MS did not result in any bacterial species with a score higher than 2.000, which secures genus and probable species identification. The highest score value was 1.885 for *Klebsiella aerogenes*, followed by 1.789 for *K. oxytoca*.

Generally, 16S-rRNA gene sequencing is one of first tools used to determine the correct bacterial species of such novel CPE isolates, and thus we considered that WGS would be a more straightforward approach to characterize the species and plasmids involved in AMR.

The MRY16-398 isolate was observed to harbor *bla*_IMP-6_, exhibited resistance to meropenem, and was positive based on the Carba NP test and DDST using SMA. Further antimicrobial susceptibility testing indicated that MRY16-398 exhibited remarkably reduced susceptibility to most β-lactam antibiotics (Table 1).

**Table 1:** Antimicrobial susceptibility testing.

Antimicrobial agent	MIC (μg/mL)/antimicrobial susceptibility	MIC breakpoint (μg/mL)^a^		
		*S*	*I*	*R*
Piperacillin	>256/R	≤16	32–64	≥128
Amoxicillin-clavulanic acid	8/S	≤8/4	8–16	≥32/16
Piperacillin-tazobactam	2/S	≤16/4	32/4–64/4	≥128/4
Cefepime	128/R	≤2	4–8	≥16
Ceftazidime	128/R	≤4	8	≥16
Imipenem	0.75/S	≤1	2	≥4
Meropenem	16/R	≤1	2	≥4
Doripenem	8/R	≤1	2	≥4
Ertapenem	>32/R	≤0.5	1	≥2
Gentamicin	8/I	≤4	8	≥16
Tobramycin	16/R	≤4	8	≥16
Amikacin	2/S	≤16	32	≥64
Minocycline	8/I	≤4	8	≥16
Ciprofloxacin	0.5/S	≤1	2	≥4
Fosfomycin	128/I	≤64	128	≥256

^a^*CLSI guideline for MIC breakpoints of Enterobacteriaceae. S, Susceptible; I, Intermediate; R, Resistant*.

### Whole-Genome Sequence Analysis of Metakosakonia sp. MRY16-398

Basic information regarding the complete chromosome and plasmid sequences of *Metakosakonia* sp. MRY16-398 is shown in Table 2. The strain possessed two plasmids, and pMRY16-398_2 was determined to be an IncN replicon plasmid, harboring multiple AMR-encoding genes including the *bla*_IMP-6_ carbapenemase-encoding gene (Table 2). The IMP-6 metallo-β-lactamase is an IMP variant with a S_214_G amino acid substitution in the catalytic domain of IMP-1, resulting in significantly diminished enzymatic activity toward imipenem but not meropenem (Oelschlaeger et al., 2005). Thus, MRY16-398 showed susceptibility to imipenem, but resistance to other carbapenems (Table 1). The *aacA4′-3* gene encoding aminoglycoside-3′′-adenylyltransferase, and the *aadA2* gene encoding streptomycin 3′′-adenylyltransferase are involved in resistance to aminoglycosides. The *tet*(A) gene could be involved in reduced susceptibility to minocycline. Comparative analyses of the MRY16-398 genome sequence including the pMRY16-398_2 plasmid are described in the following section.

**Table 2:** Whole genome information for *Metakosakonia* sp. MRY16-398.

Replicon	Nucleotide	Gene	GC%	Inc type	AMR genes	GenBank
	length (bp)	coding				ID
Chromosome	5,919,168	5,638	53.1	NA	ND	AP018756
pMRY16-398_1	224,544	239	52.8	IncFIB(K), IncFII	ND	AP018757
pMRY16-398_2	47,417	55	52.3	IncN	*aacA4′-3, aadA2, bla*_CTX-M-2_*, bla*_IMP-6_*, sul1, tet*(A)	AP018758

*NA, not available; ND, not detected*.

### Orthologous Gene Phylogenetic Analysis of *Metakosakonia* sp. MRY16-398

**FIGURE 1:**
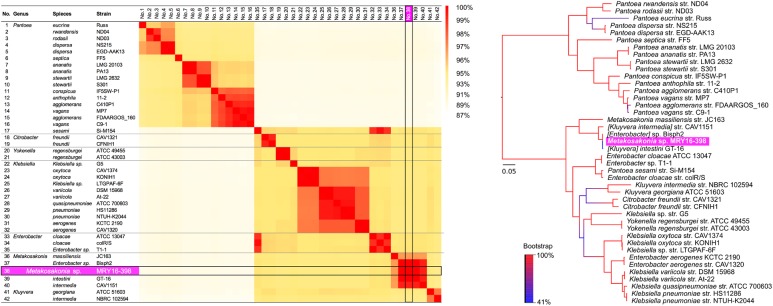
Pairwise homology sequence analysis using 479 core-gene sets from 42 strains related to MRY16-398. Maximum-likelihood phylogenetic analysis with 1,000× bootstrapping and the generation of a pairwise homology matrix were performed for the core-gene set listed in Supplementary Table S2. The MRY16-398 strain is highlighted with a bright purple background. The phylogeny and matrix distance clearly suggested that MRY16-398 is closely related to the bacterial species [*Kluyvera*] *intestini* str. GT-16 (Tetz et al., 2017), and distinct from other Enterobacteriaceae. Detailed homology % values for the matrix distances can be seen in Supplementary Table S3.

To determine the potential bacterial species of the MRY16-398 strain, we performed orthologous gene phylogenetic analysis using 41 publicly available bacterial genome sequences (on 2017/03/14), including draft genomes (see the strain list in Supplementary Table S2). Among those 42 strains including MRY16-398, 479 orthologous gene sets were extracted at ≥80% nucleotide identity, and phylogeny and matrix distance clearly suggested that MRY16-398 was closely related to the bacterial species [*Kluyvera*] *intestini* str. GT-16 (Tetz et al., 2017), which was isolated from the stomach of a patient with gastric cancer (Figure 1). Recently, the taxonomic nomenclature for [*Kluyvera*] *intestini* str. GT-16 has been re-classified into a new proposed genera, namely *Metakosakonia*, which includes *M. massiliensis* JC163 (Alnajar and Gupta, 2017). As well as the proposal, this study demonstrated GT-16 strain shows the closest lineage to *Metakosakonia*, and having a distinct lineage from the main *Kluyvera* species (*K. georgiana* and *K. intermedia*) (Figure 1). Thus, MRY16-398 was found to be a clearly distinct lineage from *Kluyvera, Pantoea*, and other well-characterized genera of the Enterobacteriaceae family (Figure 1), indicating that this clinical isolate is a novel species. Here, we tentatively classified MRY16-398 as *Metakosakonia* sp.

### Structural Comparison of pMRY16-398_2-Associated IncN Plasmids

**FIGURE 2:**
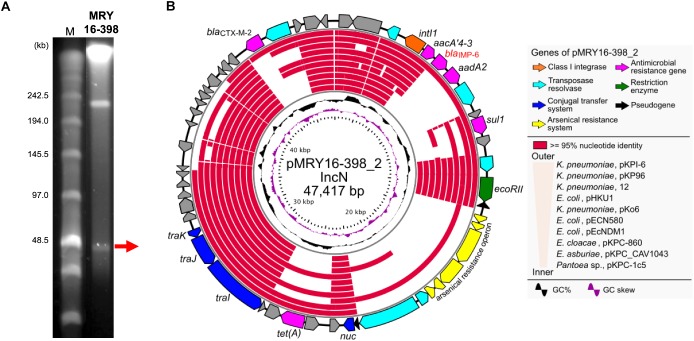
Representation of conserved gene analysis of a plasmid from *Metakosakonia* sp. MRY16-398. **(A)** Plasmids were identified by pulsed-field gel electrophoresis using an S1 nuclease-treated genomic DNA plug. **(B)** Circular representation of the plasmid pMRY16-398_2 carrying *bla*_IMP-6_, and conserved gene analysis, with comparative information for other indicated similar plasmids (Supplementary Table S4). From inward, slot 1, GC skew; slot 2, GC content; slot 3 to 12, source of IncN plasmids (see Supplementary Table S4), slot 13 and 14, open reading frames.

S1-PFGE suggested that MRY16-398 carries two plasmids (Figure 2A), and the size of both plasmids corresponded to sequencing results as well as whole genome sequencing (Table 2). An analysis of conserved genes in the pMRY16-398_2 plasmid indicated that horizontally acquired AMR genes [class 1 integron, *bla*_CTX-M-2_, and *tet*(A)] are variable in each plasmid, although IncN backbone regions remained well conserved (Figure 2B). The class 1 integron has been classified as In722 (*intI1*, *aacA4’-3*, *bla*_IMP-6_, *aadA2*, and *gc*ISKpn22) in the INTEGRALL database^2^ (Moura et al., 2009). pMRY16-398_2 shared an identical ARG profile and organization with pKPI-6 from *Klebsiella pneumoniae* KPI-6 (Figure 2B).

**FIGURE 3:**
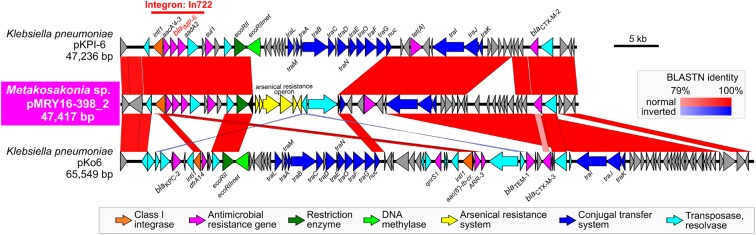
Structural comparison of the *bla*_IMP-6_-positive IncN plasmids. pMRY16-398_2 was aligned with IncN plasmid pKPI-6 (GenBank ID: AB616660) from *Klebsiella pneumoniae* KPI-6 isolated in 2007 in Japan. Homologous regions between the two are shown as red boxes with the color gradient corresponding to % homology. These plasmids carry the class 1 integron, In722 (*intI1*, *aacA4’-3*, *bla*_IMP-6_, *aadA2*, and *gc*ISKpn22), classified in the INTEGRALL database (http://integrall.bio.ua.pt/) (Moura et al., 2009), and additional *bla*_CTX-M-2_ and *tet* (A).

Pairwise alignment clearly showed that some genes involved in the conjugal transfer system have been excised and replaced with arsenical resistance proteins (Ars system) (Diorio et al., 1995) via an IS*6100*-mediated homologous recombination event (Figure 3). A mating transconjugation experiment to recipient *E. coli* showed negative plasmid transmission with pMRY16-398_2, although positive transmission was observed with a certain IncN plasmid harboring a full set of *tra* genes (data not shown). In addition to multiple AMR genes, pMRY16-398_2 appears to have lost its self-conjugation transfer ability to other bacteria, whereas it acquired arsenic resistance. This likely led to an increase in the persistence and fitness of the novel bacterial species, which is an opportunistic pathogen, in the presence of high concentrations of disinfectants in the hospital environment.

### Additional Potential AMR Genes

**FIGURE 4:**
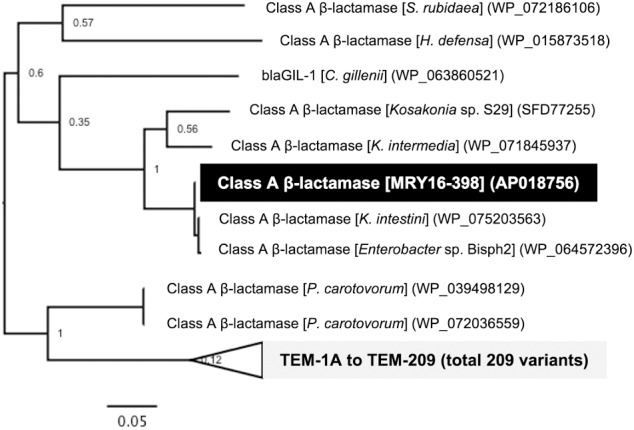
Class A β-lactamase-encoding genes in the chromosome of *Metakosakonia* sp. MRY16-398. A maximum-likelihood phylogeny among TEM-1A-related class A β-lactamases suggesting that MRY16398_50310 encodes a possible class A β-lactamase. Bootstrapping values are shown at the nodes.

A search for ARGs revealed an additional potential class A β-lactamase (MRY16398_50310), with 76% amino acid similarity to the TEM-1A variant, in the chromosomal DNA of MRY16-398 (Figure 4). A maximum-likelihood phylogeny among TEM-1A-related class A β-lactamases suggested that MRY16398_50310 is closely related to those of *Kluyvera* spp.

## Discussion

In this study, we isolated an IMP-6-producing novel Enterobacteriaceae species, *Metakosakonia* sp. strain MRY16-398, from the ascites of a diverticulitis patient. Nosocomial CPE outbreaks are generally caused by virulent pathogens; however, avirulent bacteria can cause opportunistic infection as apparent pathogens upon acquiring a notable resistance determinant. Such rare cases of avirulent or novel bacteria species have are not often documented as case reports, because conventional testing for bacterial identification are not always correct for novel species, as shown in this study. WGS comprised a straightforward approach to characterize the overall features of this isolate and its plasmids involved in AMR, and this genome sequence will be helpful for further characterization of infections caused by *Metakosakonia* sp.

A few studies related to the *Metakosakonia* genus have been reported thus far, and the most genetically related genus *Kluyvera* represents an informative reference for further discussion. *Kluyvera* spp. strains have been reported as potential pathogens in immunocompromised hosts; in addition, the *Kluyvera* genus is one source of CTX-M genes, which are thought to be the most common and important extended-spectrum β-lactamase-encoding genes (Humeniuk et al., 2002). For example, KLUA-producing *Kluyvera ascorbata* can survive for a long time in environments such as sewage and the human gut and promote drug resistance-associated gene transfer (Farmer et al., 1981). Based on a report of AMR in *Kluyvera*-related species, *bla*_GES-5_ positive, carbapenem-resistant *K. intermedia* were isolated from a hospital environment (Ribeiro et al., 2014). Further, KPC-2-producing *K. ascorbata* has been reported in a case of biliary tract infection (Wang et al., 2018). A *K. ascorbata* isolate positive for the colistin resistance gene, *mcr-1*, was identified from hospital sewage in China (Zhao and Zong, 2016). Such opportunistic pathogens including *Kluyvera* represent important multi-drug resistant bacteria in clinical settings and other environmental sources.

Here, we isolated a novel species, *Metakosakonia* sp. MRY16-398, and revealed the horizontal acquisition of the *bla*_IMP-6_ plasmid in this novel species that is rarely associated with the clinical settings. Such novel opportunistic pathogens might act as a potential reservoir/source of clinically relevant antibiotic resistance genes. In conclusion, these findings highlight the fact that bacterial identification is a crucial primary step when an isolate exhibits markedly reduced susceptibility as CPE. Moreover, continuous and comprehensive monitoring including WGS should be conducted for the detection of CPE even in nonpathogenic bacteria isolated from the clinical settings.

## Data Availability Statement

The complete, annotated genomic sequence of [*Kluyvera*] *intestini* MRY16-398 was deposited in a public database (Accession Nos. chromosome, AP018756; pMRY16-398_1, AP018757; pMRY16-398_2, AP018758). The short- and long-read sequences for DNA-Seq were deposited in the DNA Data Bank of Japan (BioProject PRJDB7098, BioSample SSUB009772, DRA accession DRA007011).

## Author Contributions

TT and AT contributed to the isolation of the IMP-6 positive *Metakosakonia* sp. strain MRY16-398. MM performed S1-PFGE analysis to detect individual plasmids. MM and SS performed antimicrobial susceptibility testing and DNA preparation for whole genome sequencing. TS and MK performed genome sequencing and the comparative genome analysis. MH performed mating transconjugation experiments. MK wrote the manuscript.

## Conflict of Interest Statement

The authors declare that the research was conducted in the absence of any commercial or financial relationships that could be construed as a potential conflict of interest.

## Footnotes

1 https://cge.cbs.dtu.dk//services/PlasmidFinder/

2 http://integrall.bio.ua.pt/
